# The Role of Microbiome and Virome in Idiopathic Pulmonary Fibrosis

**DOI:** 10.3390/biomedicines9040442

**Published:** 2021-04-20

**Authors:** Paschalis Ntolios, Vassilios Tzilas, Evangelos Bouros, Eleni Avdoula, Ioannis Karakasiliotis, Demosthenes Bouros, Paschalis Steiropoulos

**Affiliations:** 1 Department of Pneumonology, Medical School, Democritus University of Thrace, 68100 Alexandroupolis, Greece; pstirop@med.duth.gr; 2 Athens Medical Center, First Department of Pneumonology, Medical School, National and Kapodistrian University of Athens, 115 27 Athens, Greece; tzilasvasilios@gmail.com (V.T.); evangelos_@me.com (E.B.); avdoula@gmail.com (E.A.); debouros@gmail.com (D.B.); 3 Laboratory of Biology, Department of Medicine, Democritus University of Thrace, 68100 Alexandroupolis, Greece; ioakarak@med.duth.gr

**Keywords:** idiopathic pulmonary fibrosis, microbiome, virome

## Abstract

The interest in the lung microbiome and virome and their contribution to the pathogenesis, perpetuation and progression of idiopathic pulmonary fibrosis (IPF) has been increasing during the last decade. The utilization of high-throughput sequencing to detect microbial and/or viral genetic material in bronchoalveolar lavage fluid or lung tissue samples has amplified the ability to identify and quantify specific microbial and viral populations. In stable IPF, higher microbial burden is associated with worse prognosis but no specific microbe has been identified to contribute to this. Additionally, no causative relation has been established. Regarding viral infections, although in the past they have been associated with IPF, causation has not been proved. Although in the past the diagnosis of acute exacerbation of IPF (AE-IPF) was not considered in patients with overt infection, this was amended in the last few years and infection is considered a cause for exacerbation. Besides this, a higher microbial burden has been found in the lungs of patients with AE-IPF and an association with higher morbidity and mortality has been confirmed. In contrast, an association of AE-IPF with viral infection has not been established. Despite the progress during the last decade, a comprehensive knowledge of the microbiome and virome in IPF and their role in disease pathogenesis are yet elusive. Although association with disease severity, risk for progression and mortality has been established, causation has not been proven and the potential use as a biomarker or the benefits of antimicrobial therapeutic strategies are yet to be determined.

## 1. Introduction

Despite its constant exposure to the environment, the lower respiratory tract has been considered sterile for years. This notion was based on the inability of the traditional sampling and isolation methods, especially microbial cultures, to identify microbes resident in the lung [1]. The utilization of high-throughput sequencing of bacterial 16s-rRNA, has shifted the landscape and has shown high sensitivity compared to conventional microbial cultures in detecting bacteria in bronchoalveolar lavage fluid (BALF) samples [2]. The lower respiratory tract is now known to harbor complex and diverse microbial and viral communities [3,4,5,6].

Idiopathic pulmonary fibrosis (IPF) is a devastating interstitial pneumonia of unknown cause, characterized by the fibrotic distortion of normal lung architecture leading to gas exchange abnormalities and ultimately respiratory failure and death [7]. It affects older, mostly male individuals, with a median age of diagnosis of 66 years and estimates of incidence between 4.6 to 16.3 per 100,000 individuals [8]. The natural course of the disease is characterized by great variability and personalized estimations of prognosis and response to treatment present a challenge [9,10]. Nevertheless, medial survival without treatment is estimated to be 2.5–3.5 years after diagnosis, as dismal as that of many cancers [8,11].

**Figure 1:**
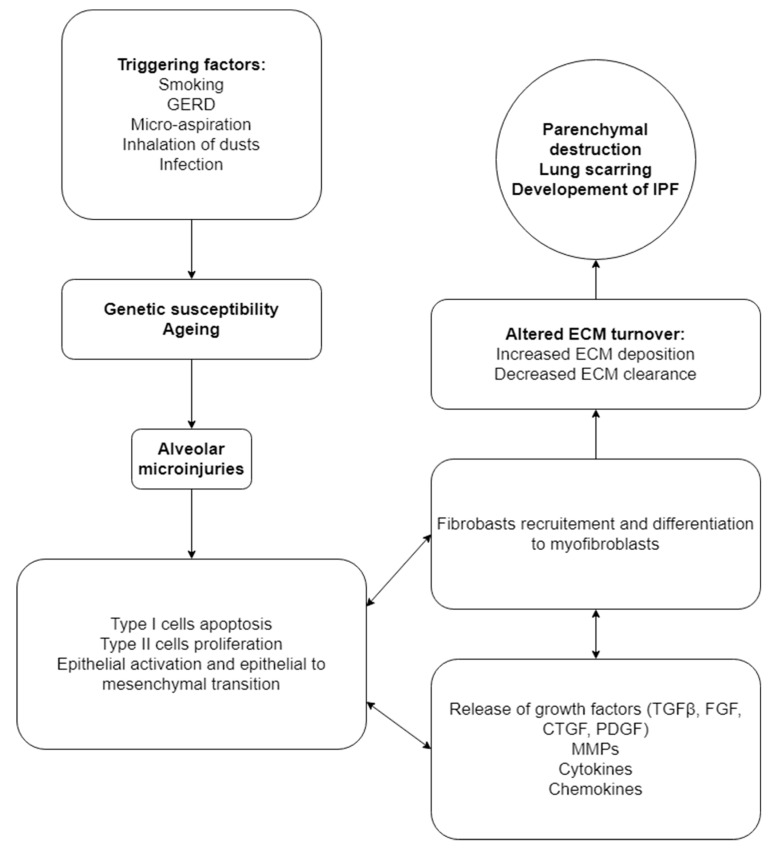
The current pathogenetic paradigm of IPF. Triggering factors imposed on genetic susceptible and ageing lung lead to aberrant wound healing responses, release of multiple mediators and fibroblast and myofibroblast activation and increased ECM deposition. GERD: Gastro-esophageal reflux disease; TGFβ: transforming growth factor β; FGF: fibroblast growth factor; CTGF: connective tissue growth factor; PDGF: platelet-Derived growth factor; MMPs: Metalloproteinases; ECM: Extra-cellular matrix; IPF; idiopathic pulmonary fibrosis.

Disease pathogenesis is yet elusive; however, the current paradigm has shifted from an inflammation-mediated disease. Instead, aberrant wound healing as a response to unknown, repetitive, low immunogenicity-generating stimuli is considered as the triggering and preserving mechanism underlying the fibrotic process [12,13]. Smoking, gastric acid reflux and micro-aspiration as well as inhaled dusts and infectious agents, especially viruses, have been traditionally implicated as precipitating factors, suspected of triggering the initiation of fibrosis in genetically susceptible individuals (Figure 1) [14]. Despite their association with sporadic or familial IPF cases, none of these factors is considered to have a causative relation to the disease.

Recently, the microbiome, the entirety of the symbiotic and pathogenic microorganisms that compose the microbial ecosystem that inhabits our body, has gained attention concerning its relation to the initiation, perpetuation and exacerbation of the fibrotic process in IPF [6,14,15]. This notion is augmented by the recent discovery that mutations in the gene encoding the mucin 5B (MUC5B), which is essential for mucociliary clearance and in host-bacterial defense, and the extracellular matrix, are associated with increased incidence of both sporadic and familial IPF [16,17].

In this review, we summarize the available evidence regarding the lung microbiome in IPF patients, the available data regarding its association with the pathogenesis and its role in the clinical course of the disease. A summary of the studies and their main results can be found in Supplement Table S1. 

## 2. Lung Microbiome and Virome in Healthy Subjects

The lung is the largest human organ in direct contact to the environment. A total epithelial and airways area of 50–75 square meters is in constant exposure to ambient air, while the total volume of air that passes through the lungs is estimated to be 10,000 L/day. In addition to that, the lungs are exposed to micro-aspiration of oropharyngeal and gastric content. Therefore, it should be expected that the lower respiratory tract is exposed to and can be the home of a plethora of microbes and viruses. Despite this, the dogma that lungs are sterile has persisted for many years and it is only during the last 10–15 years that this has changed. It is now accepted that the lower respiratory system is home to abundant and diverse populations of microbiota [1].

This new knowledge has been the result of the application of culture-independent techniques for the identification of bacteria. These techniques have focused on sequencing highly conserved loci of the bacterial genetic material, such as the 16s rRNA gene and have compared the findings with available microbial gene databases [1]. 

**Figure 2:**
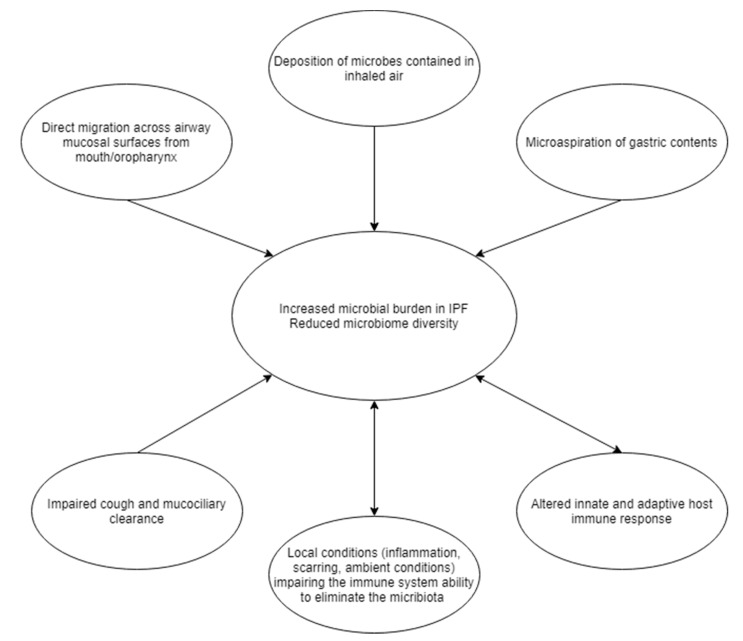
Summary of the mechanisms leading to increased microbial burden and reduced microbiota diversity in the idiopathic pulmonary fibrosis (IPF) lung. The relationship between the microbiome and local conditions as well as between the microbiome and the altered immunity is bidirectional, leading to a positive feedback loop.

The composition of the lung microbiome depends on the balance of the following three factors. First, instillation of microbiota originating from mouth, gastric content and inhaled air. Second, the ability of the lung to clear the micro-organisms, through mucocilliary clearance and cough. Last, the local environmental conditions, including oxygen partial pressure and temperature or pH fluctuations. A shift in the balance of these three factors as occurs in lung diseases can result in altered microbiome (Figure 2) [18].

In healthy subjects, the lung bacterial burden is twofold to fourfold lower compared to the oropharyngeal, but of similar composition [19]. In addition, it is similar among individuals, presenting remarkable consistency of the dominant taxonomic units [20]. In previously published studies, the most prevalent phyla in the normal lung were reported to be *Bacteroidetes*, *Firmicutes*, *Proteobacteria* and *Actinobacteria* [21,22,23]. The most prominent genera were *Prevotella, Streptococcus* and *Veillonella* [21,22,23]. In a healthy single subject, these microbiota are found throughout the respiratory tract with a trend of reduction in abundance over the lower areas [21]. The lack of spatial heterogeneity among lung regions and lobes and the associated varying local oxygen, pH and temperature conditions, raises the possibility that the lung microbiome in health depends mostly on the microbial instillation and elimination turnover [21]. Interestingly, the consistency of the lung microbiome perseveres among various geographic loci. This has been demonstrated by comparisons in the population of different cities in the USA, which resulted in no distinct clustering. On top of that, their microbiome resembled that of healthy British volunteers [22,24,25]. Interestingly, this is in contrast to the finding that microbiome of the normal gastro-intestinal tract shows important variation depending on the geographic region [26].

Unlike the microbiome, the lung virome has been less well studied. It is stated however that the virome exhibits considerable variation and can be is considered as a triggering factor for many lung diseases [27].

## 3. Lung Microbiome in Stable IPF

Historically, due to the prevailing dogma that lungs are sterile, resident microbial populations have not seen the same attention as viral infections as possible triggers of disease. It is now known that the lungs are not sterile and that the IPF lung specifically exhibits a distinct microbial flora compared to the normal lung [14]. Despite the subpar capability of traditional microbial cultures to identify lung microbial populations, a culture-based study by Richter et al. has found that in IPF lung microbial burden is higher and species such as *Haemophilus influenzae*, *Haemophilus parainfluenzae*, *Streptococcus pneumoniae*, *Moraxella catarrhalis*, *Pseudomonas aeruginosa* and *Proteus mirabilis* have been isolated in cultures from BALF of 8 out of 22 patients with IPF [28]. Notably, in this study most of the IPF patients were under immunosuppressive therapy at the time.

Since the commencement of the employment of culture-independent techniques for the detection of microbial 16s-rRNA, organisms normally present in the oropharynx as well as organisms unable to be identified by microbial cultures, including *Neisseria*, *Actinobacteria* and *Streptococcus* species, have been detected in IPF patients [29]. Garzoni et al. also used ultra-deep 16S rRNA gene sequencing to investigate the microbiota of either patients with idiopathic interstitial pneumonia (IIP) or Sarcoidosis, normal controls or patients with *Pneumocystis Jirovecii* infection [30]. The microbiota in the majority (90%) of subjects consisted of *Prevotellaceae*, *Streptococcaceae* and *Acidaminococcaceae*, with no significant differences among patients and healthy controls [30].

The Correlating Outcomes with biochemical Markers to Estimate Time-progression in idiopathic pulmonary fibrosis (COMET) multicenter cohort study was an effort to identify unique microbial signatures and investigate their association with disease progression [31]. The authors used BAL samples from retrospectively identified IPF patients to evaluate the potential contribution of the lung microbiome in disease progression, in a first effort to establish a causal association between IPF and the microbiome [31]. The study included 55 IPF patients with mean forced vital capacity (FVC) 70.1% predicted and mean diffusion lung capacity for carbon monoxide (DLco) 42.3% predicted. All participants were prospectively followed-up for up to 80 weeks, in 16-weeks intervals. BALF was sequenced for the genome of bacteria and the most prevalent species detected were *Prevotella*, *Veillonella* and *Cronobacter* spp. More importantly, the presence of a specific *Streptococcus* or *Staphylococcus* species, as identified by their Operational Taxonomic Unit (OTU), was associated with faster disease progression, even after adjusting for confounding factors [31]. However, these OTUs were found in less than half of the patients that were included in the study and despite their association with faster progression, a causal relationship with the development of the disease could not be established [31].

Molyneaux et al. published a large study in 2014 that included 65 patients with IPF, 27 healthy controls and 17 patients with Chronic Obstructive Pulmonary Disease (COPD) [24]. The authors tried to elucidate the role of the bacteria in the pathogenesis and progression of IPF. The abundance of bacteria in the IPF lung was confirmed, with a bacterial load twofold higher in the BALF of these patients compared with both healthy controls and COPD patients [24]. Additionally, higher bacterial load was associated with increased risk for disease progression at 6 months and mortality (HR 4.59) and additionally with the presence s35705950 polymorphism of the MUC5B mucin gene, a known predisposing factor for the development of IPF [24]. In the same study, the abundance of the OTUs for *Veillonella*, *Neisseria*, *Streptococcus* and *Haemophilus* spp. was consistently associated with IPF. No significant differences were found in the BAL of healthy control subjects and patients with COPD. The authors hypothesized that, when taking into consideration the results of the COMET study, it is the increased bacterial burden and not specific populations that can predict disease progression and mortality [24,31].

Most recently, a study compared the microbiome of patients with IPF to that of patients with chronic hypersensitivity pneumonitis (cHP). The results suggested that the bacterial load in cHP is higher than normal controls, however it is significantly lower compared to that of subjects with IPF [32]. Moreover, bacterial load was not associated with survival in the cHP group, in contrast to the IPF group where, in line with the evidence from previous studies, higher bacterial burden was associated with higher mortality risk [32]. Regarding specific microbial populations, the phylum *Firmicutes* was more prevalent in IPF, while in cHP *Proteobacteria* were more abundant. At genus level, cHP had higher *Staphylococcus* burden while in IPF *Actinomyces* and *Veillonella* load was increased [32].

Impaired lung microbiota diversity has also been implicated in the progression of IPF. In a study by Takahashi et al., increases in BALf of the *Streptococcaceae*, *Veillonellaceae*, and *Prevotellaceae* families and a decrease in the phylum *Proteobacteria* were involved in the reduction of diversity and correlated with low FVC and decreased 6MWT distance, as well as increased serum surfactant protein D (SP-D) and LDH [33]. The results were confirmed using an experimental fibrosis model fibrosis [33]. However, the lack of a control group diminishes the value of these results.

In contrast to the utilization of BAL for the detection of bacterial populations in the IPF lung, Valenzi et al. used a different approach [34]. This study group used genetic sequencing to detect bacterial 16s rRNA in parenchymal tissue samples from patients undergoing lung transplantation for IPF, COPD, connective-tissue disease-associated ILD (CTD-ILD) and cystic fibrosis (CF). In addition, they obtained tissue samples from donor lungs that were unsuitable for transplantation [34]. The study included 62 patients with end-stage IPF that were undergoing lung transplantation and identified that basilar lung parenchymal samples had consistently decreased bacterial burden compared with airways samples [34]. In addition, a subgroup of patients demonstrating higher bacterial load was more prone to worse clinical outcomes, including disease exacerbation and death [34]. The authors commented that their findings suggest that BAL studies do not identify parenchymal but airways microbiota, but nevertheless patients with higher bacterial burden have worse prognosis [34]. This finding is in agreement with previous studies that utilized BALF samples [24,31,32,33,34].

## 4. Lung Virome in Stable IPF

Unlike microbial detection that required technologically advanced techniques, viral detection through serology presented a more accessible option. Viral infections have been long considered as a potential precipitating factor for the development of IPF in susceptible individuals. Vergnon et al. have studied the association of the human herpes viruses (HHVs) as early as in 1984 [35]. In this study, 10 patients with IPF out of a total of 13 had increased serum antibody titles for the Epstein-Barr virus (EBV), in contrast to none of the 12 patients from the control group, with a diagnosis of other interstitial lung disease (ILD) [35]. Increased prevalence of EBV in lung biopsy samples and BALF of patients with IPF when compared to normal controls has been confirmed in many following studies, however, none of them managed to confirm a causal relationship [36,37,38,39].

The hepatitis C virus (HCV) has also been associated with IPF, although inconsistently. In one study, serum antibodies against HCV have been found in 28.8% of 66 IPF patients compared to 3.66% of 9464 age and gender-matched controls [40]. In addition, another study has found that in patients with known HCV infection, IPF has increased 10 and 20-year cumulative incidence (0.3 to 0.9% among 6150 patients) compared to patients infected with the hepatitis B virus (HBV) (zero cases in 2050 patients) [41]. In the same study, patients that were heavy smokers, older than 55 years and had liver cirrhosis were at higher risk for developing IPF, raising the possibility that one of these confounding factors was responsible for the development of IPF [41]. Contrary to these studies, Irving et al. found that in 62 patients with IPF, HCV infection was no more prevalent compared to the general population [42].

Similar to the study by Valenzi et al. [34], a study by Yin et al. utilized next-generation RNA sequencing to detect viral RNA abundance in lung tissue samples from 28 patients with IPF and 20 control subjects that had undergone surgical lung biopsy [43]. The abundance of viral RNA belonging to 740 viruses was quantified and key sequencing results were confirmed for specific viruses (EBV, HCV, herpesvirus saimiri and HERV-K) by real-time quantitative polymerase chain reaction (qPCR). Despite the confirmation of sporadic presence of viral RNA in the tissue specimens, there were no significant differences between IPF and control lung regarding the abundance of viral RNA [43]. This study was the first to use RNA sequencing to detect viral signatures, in contrast to the previously used serologic tests.

## 5. Lung Microbiome in Acute Exacerbation of IPF

The latest revision of the diagnostic criteria for the acute exacerbation of IPF (AE-IPF) added overt infection as potential trigger of AE-IPF [44]. In the past, active infection was considered to rule out the diagnosis of AE-IPF [45]. Occult infection has always been on the focus as a possible etiology for AE-IPF.

Regarding bacterial burden and its contribution to AE-IPF, Molyneaux et al. have utilized bronchoscopy with BAL and DNA extraction and found that patients with AE-IPF had up to four times higher bacterial load when compared to stable IPF patients [46]. Additionally, a shift in the microbial OTUs compared to stable disease was detected by 16S rRNA sequencing. A significant increase was noticed for *Proteobacteria*, *Campylobacter* spp. and *Stenotrophomona* spp., while a significant decrease was found in *Veillonella* spp. and *Campylobacter* spp. The authors concluded that the microbial burden might have a causative role in the pathogenesis of AE-IPF [46]. Notably, the study used the old definition for acute exacerbation that required exclusion of any overt infection as a cause for the exacerbation [45].

More recently, Weng et al. examined the sputum cultures of 170 patients with AE-IPF [47]. Gram-negative bacteria were found to dominate, consisting the 89% of the 38 different strains that have been found. Specifically, *Klebsiella pneumonia* accounted for 26%, *Mycobacteria tuberculosis* for 21% and *Acinetobacter baumannii* for 10% of the total strains [47].

Pre-clinical studies in mouse models have also been used to test the hypothesis that microbial infection can be responsible for disease exacerbation. *Streptococcus pneumoniae* has been found to induce exacerbation of fibrosis [48]. Moreover, in a germ-free mouse model, bleomycin induced fibrosis of equal severity to both germ-free and conventional mice; however, the germ-free mice were protected against mortality [49]. These data are in accordance to clinical observations, although the discrepancy of the mouse model of fibrosis to IPF must be taken into consideration.

## 6. Lung Virome in Acute Exacerbation of IPF

Viral infections by serologic studies have also been investigated concerning their association with the development and progression of IPF, as well as with acute exacerbations [50]. In a study that utilized multiplex polymerase chain reaction, pan-viral microarray and high-throughput cDNA sequencing, viral nucleic acid was detected in the BALF of 19 out of 43 cases of AE-IPF, compared to none in the stable disease group [51]. Interestingly, the torque teno virus (TTV) that was present in the BA BALF of 12 patients of this group has been associated with worse survival in the past [51,52]. Many other viruses have also been implicated in the pathogenesis of AE-IPF. Weng D et al. identified 57 different viruses in the nasopharyngeal swabs of 18 out of 30 patients with AE-IPF compared to 13 out of 30 of patients with stable disease [47]. The most prominent viruses in the acute exacerbation group were HHV as well as influenza virus A [47].

## 7. Microbial Involvement and Host Response in IPF Progression

Despite the progress in the identification of the microbiome and its association with IPF, it is still unclear whether there is a causal relation or if the altered microbiome is the result of the disease.

Infection is carrying significant morbidity and mortality in IPF patients and is a known cause of acute exacerbation of the disease [44]. In patients with IPF, prophylactic treatment with co-trimoxazole has been utilized and has resulted in the reduction of both infections and mortality [53]. Moreover, the failure and the premature interruption of the prednisone/Azathioprine/N-Acetylcysteine arm of the PANTHER trial has taught us that immunosuppression has deleterious effects for individuals with IPF [54]. Furthermore, during the last decade, genetic studies have documented that polymorphisms in genes linked to host immunity can lead to susceptibility for the development of IPF and influence the prognosis [55]. Specifically, the polymorphisms in the MUC5B and the Toll-interacting protein (TOLLIP) encoding genes have been linked to increased susceptibility for developing IPF and to altered host immune response [16,56,57]. MUC5B is encoding an essential protein for the normal macrophage function and mucocilliary clearance, while TOLLIP is related to the regulation of the innate immune response that is mediated through pattern-recognition Toll-like receptors [56,57,58].

Despite the above-mentioned relations, a causative effect of the host-immune interactions and the development of IPF has not been established yet. This potential association was studied by Molyneaux at al., who tried to explore the relation between the peripheral whole-blood transcriptome, the respiratory microbiome and the presence of MUC5B and TOLLIP polymorphisms in patients with IPF [59]. More specifically, BALF and peripheral blood were collected from 60 patients with IPF and 20 healthy subjects. All of the participants were followed-up for up to 12 months. The authors used network analysis of gene expression data on BALF and peripheral blood and identified two gene modules that were strongly associated with the diagnosis of IPF, bacterial burden in BALF and the presence of specific microbes, as determined by their OTUs [59]. The same genes were associated with BALF and peripheral blood neutrophilia [59]. Within the modules, genes that were related to host immune response were NLRC4, PGLYRP1, MMP9 and DEFA4 along with two genes encoding specific antimicrobial peptides [59]. Among them, many were associated with survival and were consistently over-expressed longitudinally [59]. These findings suggest that the respiratory microbiome in IPF incites a host defense response that is maintained throughout the disease course, raising the possibility that this could act as persisting stimulus leading to repetitive epithelial injury.

Another study by Huang et al. attempted to explore the connection of microbial interaction and host immune response to the progression-free survival (PFS), in vitro fibroblasts function and leukocytes phenotypes [60]. Sixty-eight IPF patients from the COMET study were recruited [31]. The authors demonstrated that down-regulation of the host immune response by relative inhibition of 11 pathways, including nucleotide-binding oligomerization domain (NOD)-, Toll-, and RIG1-like receptor, was associated with worse PFS [60]. Ten out of the 11 immune response pathways that were associated with worse PFS were also correlated with microbial diversity and increased abundance of specific OTUs [60]. Specifically, the decrease of the activity of the (NOD)-like receptor pathway was associated with increased abundance of *Streptococcus* sp. and poor PFS. Abundance of *Staphylococcus* and *Prevotela* sp. was also associated with worse PFS, decreased activation of immune defense pathways and overexpression of Toll-like receptor 9 (TLR-9) in peripheral blood mononuclear cells (PBMC) [60]. The authors concluded that hots-immune interactions are key for disease progression in IPF.

## 8. Lung Microbiome as a Treatment Target in IPF

A double-blind, randomized placebo-controlled trial by Varney et al., published in 2008, compared co-trimoxazole to placebo in patients with progressive fibrotic IIP [61]. Patients that received co-trimoxazole exhibited improved 6MWT distance with reduced oxygen desaturation, improved FVC, reduced ground glass opacities on HRCT and better quality of life (QoL) scores after 12 months of treatment [61]. 

In a larger study, 181 patients with fibrotic IIP, mostly IPF, were assigned to either co-trimoxazole or placebo. After 12 months, co-trimoxazole had no effect on FVC, DLco, 6MWT or on dyspnea scores but improved quality of life markers and all-cause mortality on an intention-to-treat analysis [53]. To further investigate their findings, the authors planned a new double-blind, placebo-controlled, randomized, multicenter trial aiming in recruiting 330 patients with IPF to test the hypothesis that addition of co-trimoxazole to standard treatment can increase time to death, lung transplant or first non-elective hospitalization [62]. This trial is still ongoing.

Another anti-microbial agent that has been studied in IPF is doxycycline. Mishra et al. recruited six patients with IPF in an open-label, prospective study to test the efficacy of long-term doxycycline as an inhibitor of metalloproteinases (MMPs). BALF samples were acquired before the initiation of doxycycline and while under treatment and MMPs levels were compared to those of healthy subjects [63]. Additionally, functional (FVC, 6MWT) and QoL indices (Saint George’s Respiratory Questionnaire—SGRQ) were recorded. Doxycycline treatment was associated with non-significant trend towards improvement of FVC and 6MWT, while significant improvement was noted in the SGRQ [63]. In addition, the level of MMP3, MMP9, tissue inhibitor of metalloproteinases (TIMP-1) and vascular endothelial growth factor (VEGF) were significantly higher in the BALF of IPF patients compared to controls and were reduced to control subjects’ level after doxycycline administration [63].

In addition to the data above, the association of the microbiome to disease progression and to the immune system dysregulation in IPF, has led to the hypothesis that treatment with antibiotics could modulate the underlying microbiome of patients with IPF towards that of the normal lung [24,31,46,59,60]. A favorable effect of this approach has been documented in other chronic lung diseases [1]. The CleanUP-IPF is an undergoing multi-center, pragmatic, open-label, randomized trial of antimicrobial therapy of patients with IPF [64]. The study aims in comparing standard of care vs standard of care plus anti-microbial therapy with either co-trimoxazole or doxycycline and the primary end-point is the first non-elective, respiratory hospitalization or all-cause mortality [64].

## 9. The “Gut-Lung” Axis in Lung Disease and IPF

The comparison of the lung microbiome with that of other systems has been extensively studied and the association with lung diseases such as asthma, COPD and lower respiratory tract infections (LRTIs) is known [65]. The gastrointestinal tract is known to harbor the largest microbial biomass of the human body [66]. At the phylum level, gut and lung microbiomes are similar and include *Bacteroidetes* and *Firmicutes*, however there are differences at species level [67,68].

The disruption of gut microbiome is known to precipitate diseases of the gastrointestinal tract, but also of other systems [69]. The cross-talk between the gut microbiome and the lungs has been demonstrated [70]. There is increasing evidence that this “gut-lung” axis is part of the pathogenesis of many lung diseases, including COPD, asthma, cystic fibrosis and LRTI [71]. Moreover, alterations in the gut microbiota have been shown to modulate immune system responses in the lung by various mechanisms, including through regulatory T cells (Tregs), TLRs and inflammatory cytokines [72,73,74,75].

Regarding IPF, there is no evidence of a direct involvement of the gut microbiota in the pathogenesis and progression of the disease. However, taking into consideration the increased frequency of Gastro-esophageal reflux in IPF patients, indirect implication of the gut microbiota can be presumed [76,77]. Additionally, the protective function of the gut microbiome against respiratory infections in pre-clinical and clinical studies has been demonstrated [78,79,80,81]. This might be of benefit for IPF patients, since the contribution of lung infection to morbidity and mortality of these subjects is well-established [44].

## 10. COVID-19 and IPF

The coronovirus disease 2019 (COVID-19) pandemic caused by the severe acute respiratory syndrome coronovirus 2 (SARS-CoV2-2) has infected millions of people and has caused millions of deaths during the last 20 months [82]. The clinical presentation ranges from asymptomatic patients to patients with ARDS. Taking into consideration the possible association of IPF with viral infections, the spread of COVID-19 disease and that pulmonary fibrosis is a known sequalae of ARDS, suspicion has been raised that fibrotic lung disease could develop following SARS-CoV-2 infection [83,84,85]. 

Two studies have tried to address the issue of long-term fibrosis attributed to COVID-19. A study by Mandal et al., followed-up patients with persisting abnormalities on blood samples or chest-X-ray (CXR) 47–59 days after their discharge [86]. Of the 384 patients included in the study, 9% had a deteriorating CXR on follow-up [86]. 

Another study by Myall et al., screened 837 patients that have been hospitalized with COVID-19 [85]. In 35 patients, evidence of ILD, especially Organizing Pneumonia was found. Most of these patients were successfully treated with steroids [85].

Apart from the possibility to induce a fibrotic response in the lungs, COVID-19 can be potentially lethal for subjects who already suffer from fibrotic lung disease. It is possible that patients with IPF might exhibit an increase in hospitalization and adverse events due to COVID-19. Surprisingly, a study by Papiris et al., including 550 patients with IPF demonstrated that they had lower rate of SARS-CoV-2 infection compared to general population and additionally fewer hospitalizations due to other lower respiratory tract infection and deaths were reported [87]. These results were attributed to the beneficial impact of social distancing during the first pandemic wave in Greece [87].

Collectively, these data suggest that to this day, there is no evidence that COVID-19 can cause an IPF like clinical disease characterized by relentless progression and additionally adds credibility to the notion that subjects with IPF should practice social distancing during the winter months.

## 11. Conclusions

The last decade has seen the advancements in molecular sequencing techniques revolutionizing the detection of microbial populations in the lung. Furthermore, the current understanding of IPF pathogenesis is based on the assumption that aberrant would healing as a response to unknown, repetitive, low immunogenicity-generating stimuli is the triggering and preserving mechanism underlying the fibrotic process. The role of microbiome in IPF is not yet fully elucidated, however it is clear that infection is a strong inducer of morbidity and mortality in these patients. In light of this, it is possible that the microbiome contributes to the triggering and preservation of the epithelial damage. Further studies focusing on the investigation of a causal relationship, on identifying microbial biomarkers and on exploratory therapeutic approaches are needed.

## Data Availability

The study did not report new data.

## Supplementary Materials

The following are available online at https://www.mdpi.com/article/10.3390/biomedicines9040442/s1, Table S1: Studies investigating the microbiome and virome in IPF and their main results and microbial populations.
