# Supplementary material for: The Role of Microbiome and Virome in Idiopathic Pulmonary Fibrosis
Source: Biomedicines. 2021 Apr 20;9(4):442. doi: 10.3390/biomedicines9040442 (PMC8074588; doi:10.3390/biomedicines9040442)
Supplement: Supplementary file 1 [file biomedicines-09-00442-s001.zip › biomedicines-1147228-supplementary.pdf]

Studies investigating the microbiome and virome in IPF and their main results and microbial populations			
**Study**	**Method**	**Disease**	**Main results related to IPF/Microbial population in IPF patients**
Richter et al (28)	Quantitative cultures of BAL	IPF<br>ANCA-related Cocciomatosis (Wegener granulomatosis)	BAL cultures positive in 8/22 IPF patients: *Haemophilus influenzae*<br>Haemophilus parainfluenzae<br>*Moraxella catarrhalis*<br>*Pseudomonas aeruginosa*<br>*Proteus mirabilis*<br>*Streptococcus pneumoniae*
Friaza et al (29)	16S-rDNA PCR - denaturing gradient gel electrophoresis - bacterial band identification in BAL	IPF	Identified organisms normally present in the oropharynx<br>Bacterial sequences corresponding to the *Streptococcus*, *Neisseria* and *Actinobacterium* genera.<br>*Pneumocystis jirovecii* may impair bacterial colonization of the airways
Garzoni et al (30)	16s-rRNA gene sequencing of BAL	IIP<br>Sarcoidosis<br>Normal controls<br>PCP	No significant differences between ILD and healthy controls.<br>*Prevotellaceae, Streptococcaceae* and *Acidaminococcaceae*
Han et al (31)	454 pyrosequencing was used to assign OTUs in BAL	IPF	*Prevotella, Veillonella* and *Cronobacter spp*<br>*Streptococcus* OTU1345<br>*Staphylococcus* OTU1348
Molyneaux et al (24)	16S rRNA qPCR and pyrosequencing in BAL	IPF	Patients with IPF had double the burden of bacteria in BAL fluid<br>Baseline bacterial burden predicted the rate and risk of death and was associated independently with the rs35705950 polymorphism of the MUC5B mucin gene<br>*Veillonella*, *Neisseria*, *Streptococcus* and *Haemophilus* spp were more abundant in IPF than controls
Invernizzi et al (32)	Amplification of the V4 hypervariable region of the 16S rRNA gene in BAL	IPF vs HP	Distinct differences in the microbial profiles were evident in the lower airways of subjects with CHP and IPF<br><br>IPF *Firmicutes* phyla*, Actinomyces* and *Veillonella* genera<br>HP *Proteobacteria* phyla*, Actinomyces* and *Veillonella* genera
Takahashi et al (33)	Amplification of the V2–4-8 and V3–6, 7–9 hypervariable region of the 16S rRNA gene in BAL	IPF<br>Bleomycin-treated mice	The most prevalent lung phyla were *Firmicutes*, *Proteobacteria* and *Bacteroidetes*.<br>Decreased microbial diversity was associated with low FVC and early mortality.<br>Diversity and abundance of *Firmicutes*, *Streptococcaceae*, and *Veillonellaceae* were significantly associated with FVC, 6-min walk distance, and serum surfactant protein D<br>Bleomycin-induced lung fibrosis resulted in decrease of diversity and alteration of microbiota
Valenzi et al (34)	PCR amplification and sequencing of the 16S rRNA gene in parenchymal and airways tissue samples	IPF<br>CTD-ILD<br>CF<br>COPD<br>Normal Subjeccts	Airway-based samples had higher bacterial load compared to parenchymal<br>Bacterial load was lower in IPF compared to CF and controls
Calabrese et al (36)	Nucleic acids were extracted from fresh lung tissues using a modified RNAzol method RT-PCR, PCR or nested-PCR were used to detect principal respiratory viral genomes	IPF<br>Normal subjects	Higher frequency of virus positive cases was found in IPF<br>Only herpes virus genomes were detected<br>Viral cases showed higher mPAP, poorer performance in the six minute walking test and higher frequency of primary graft dysfunction

Yin et al (43)	Surgical lung biopsies<br>viral RNA qPCR	IPF	740 viruses were quantified<br>Key sequencing results were confirmed for specific viruses (EBV, HCV, herpesvirus saimiri and HERV-K)
Molyneaux et al (46)	DNA extraction from BAL<br>A hyper-variable region of the 16S ribosomal RNA gene (16S rRNA) was amplified, quantified and pyrosequenced	IPF	Increased burden 4-fold<br>Shift from stable disease OTUs<br>A significant increase was noticed for *Proteobacteria*, *Campylobacter spp*. and *Stenotrophomonas spp*., while a significant decrease was found in *Veillonella spp*. and *Campylobacter spp*
Weng et al (47)	Antiviral/bacterial IgM in Sputum cultures<br>RNA sequences of pathogens in nasopharyngeal swab of IPF patients were detected by PathChip	IPF	Thirty-eight different bacterial strains were detected in IPF patient sputum - 89% were gram-negative bacteria:<br>*Klebsiella pneumonia* 26%<br>*Mycobacteria tuberculosis* 21%<br>*Acinetobacter baumannii* 10%<br>Fifty-seven different viruses were detected in nasopharyngeal swabs of IPF patients.
O'Dwyer et al (49)	DNA extraction from mouse tissue and human BAL<br>Droplet digital PCR for the 16S rRNA gene	IPF<br>Bleomycin-treated mice	Lung Bacterial Burden Predicts Disease Progression in IPF<br>Bleomycin induces fibrosis of equal severity to both germ-free and conventional mice; however the germ-free mice were protected against mortality
Wootton et al (51)	Multiplex PCR, pan-viral microarray and high-throughput cDNA sequencing in BAL	IPF<br>AE-IPF<br>ALI	Viral nucleic acid was detected in cases of AE-IPF but not in stable disease<br>Evidence of viral infection with herpes simplex virus, Epstein-Barr virus and torque teno virus (TTV) in patients with acute exacerbation<br>TTV infection was significantly more common in patients with acute exacerbation

BAL Bronchoalveolar lavage; IPF Idiopathic Pulmonary Fibrosis; ANCA Antineutrophil Cytoplasmic Antibody; PCR Polymerase Chain Reaction; IIP Idiopathic Interstitial Pneumonia; PCP Pneumocystis Jirovecii Pneumonia; ILD Interstitial Lung Disease; OTU Operational Taxonomic Unit; qPCR Quantitative Polymerase Chain Reaction; CHP Chronic Hypersensitivity Pneumonitis; FVC Forced Vital Capacity; CTD-ILD Connective Tissue Disease – Interstitial Ling Disease; CF Cystic Fibrosis; COPD Chronic Obstructive Lung Disease; mPAP mean Pulmonary Artery Pressure; EBV Epstein-Barr Virus; HCV Hepatitis C Virus; HERV-K Human Endogenous Retrovirus type K;
